# Supplementary material for: A cerebellar internal model calibrates a feedback controller involved in sensorimotor control
Source: Nat Commun. 2021 Nov 18;12:6694. doi: 10.1038/s41467-021-26988-0 (PMC8602262; doi:10.1038/s41467-021-26988-0)
Supplement: Supplementary file 1 — Supplementary Information [file 41467_2021_26988_MOESM1_ESM.pdf]

Supplementary Information for

A cerebellar internal model calibrates a feedback controller involved in sensorimotor control

Daniil A. Markov, Luigi Petrucco, Andreas M. Kist and Ruben Portugues*

*Corresponding author. Email: ruben.portugues@tum.de

This PDF file includes:

Supplementary Figure 1: Behavior of the feedback control model of acute reaction 2
Supplementary Figure 2: Anatomical location of sensory- and motor-related ROIs is consistent across fish 3
Supplementary Figure 3: Treatment of *Tg(PC:epNtr-tagRFP)* larvae with metronidazole ablates the PCs 4
Supplementary Figure 4: Acute reaction is not impaired after PC ablation 5
Supplementary Figure 5: Long-term adaptation effects are detectable in the light-sheet functional imaging experiment 6
Supplementary Figure 6: Activity of *0-0+* ROIs cannot be explained by behavior 7
Supplementary Figure 7: *0-0+* ROIs represent a spatially distributed subpopulation of PCs 8

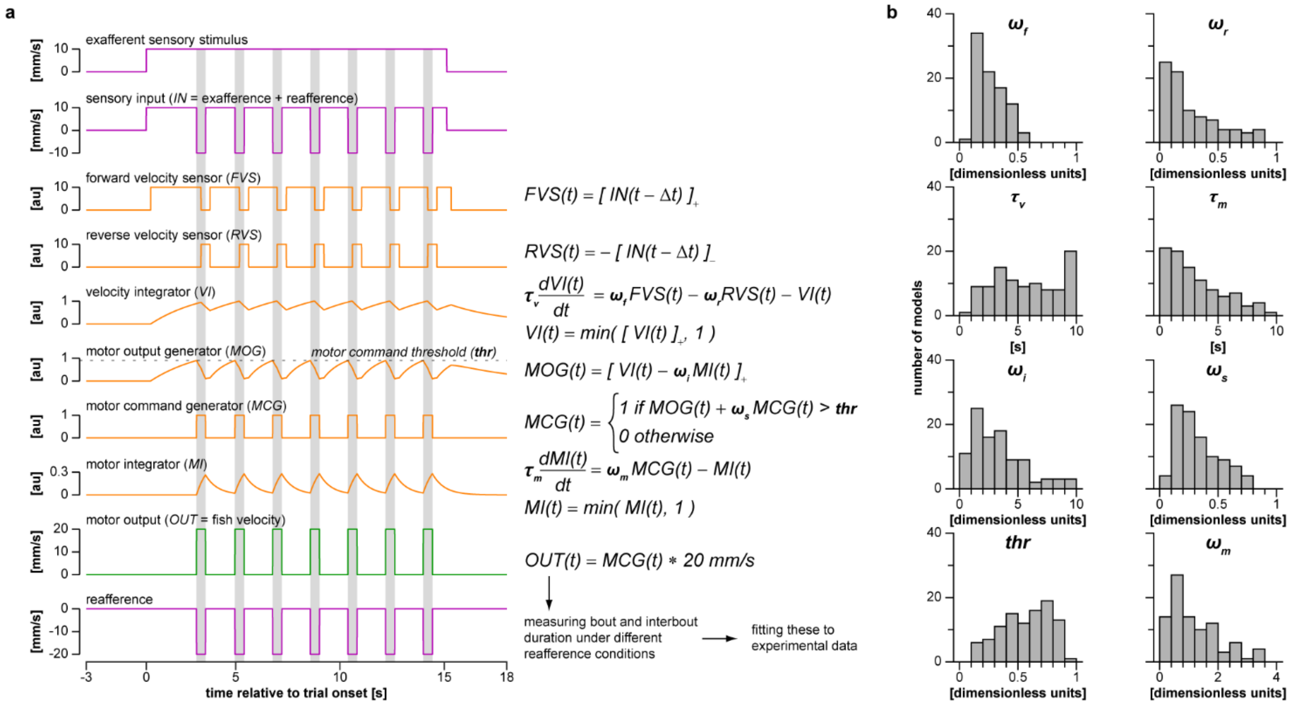

Supplementary Figure 1: Behavior of the feedback control model of acute reaction

(a) Behavior of the model in an example trial, where reafference condition was set to normal for simplicity. Magenta traces represent input of the model (grating speed, where positive values correspond to motion in a caudal to rostral direction); green trace represents output of the model (binary swimming variable representing current swimming velocity); orange traces represent output of respective nodes. Vertical shaded bars indicate swimming bouts performed by the model in this trial. Seven small Greek letters and thr denote eight parameters of the model:

- ω_f - output weigh of forward velocity sensor
- ω_r - output weigh of reverse velocity sensor
- τ_v - time constant of the velocity integrator
- ω_i - inhibitory output weigh of the motor integrator
- ω_s - feedforward self-excitation weight of the motor command generator
- thr - threshold of the motor output command
- ω_m - input weight of the motor integrator
- τ_m - time constant of the motor integrator

Δt denotes sensory processing delay of 220 ms, t - current time point. $[x]_+ = max(x, 0)$ - positive rectification of x; $[x]_- = min(x, 0)$ - negative rectification of x; $min(x, 1)$ - saturation of x at 1.

(b) Distributions of eight fitted parameter solutions across models, each fitted to an individual larva (N = 100). A few outliers defined independently for each distribution as parameter values that are more than three scaled median absolute deviations were removed for clarity of the histograms.

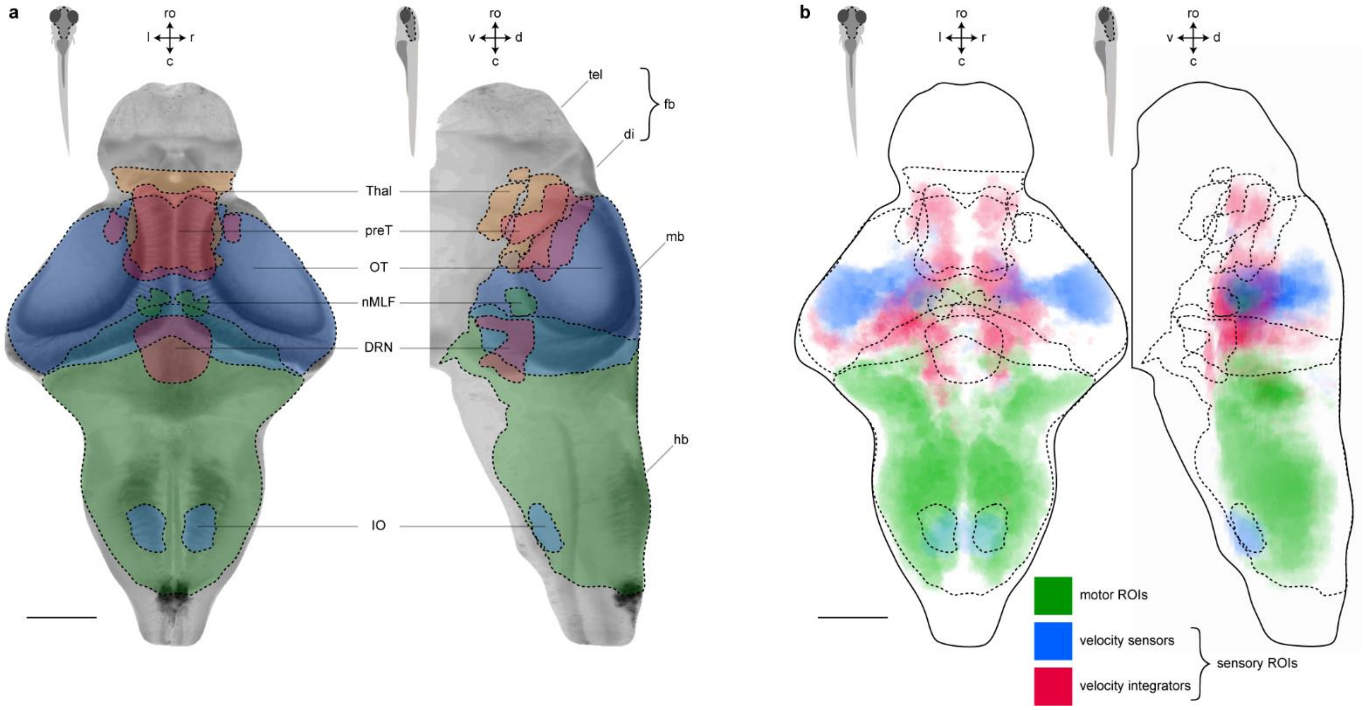

Supplementary Figure 2: Anatomical location of sensory- and motor-related ROIs is consistent across fish

(a) Selected anatomical regions in the larval zebrafish reference brain. tel - telencephalon, di - diencephalon, fb - forebrain, mb - midbrain, hb - hindbrain. Presented images are maximum projections along dorsoventral or lateral axis. ro - rostral direction, l - left, r - right, c - caudal, d - dorsal, v - ventral; scale bars: 100 µm. Colored areas depict brain regions that contained large fractions of motor- and sensory-related ROIs (Fig. 3): Thal - thalamus, preT - pretectum, OT - optic tectum, nMLF - nucleus of the medial longitudinal fascicle, DRN - dorsal raphe nucleus and surrounding reticular formation, IO - inferior olive. These anatomical regions were annotated in the Z-Brain atlas[70] and registered to our reference brain.

(b) Brain areas that consistently contain motor ROIs (green), sensory ROIs with short time constants (blue) and with long time constants (red) across imaged larvae (N = 6; see Functional imaging data analysis in Methods for details). Presented images are sum projections along the dorsoventral or lateral axis. Note that dorsal rostrolateral parts of the midbrain do not contain colored areas because these regions were blocked from the scanning laser beams by the eye-protecting screens shown in Fig. 3a.

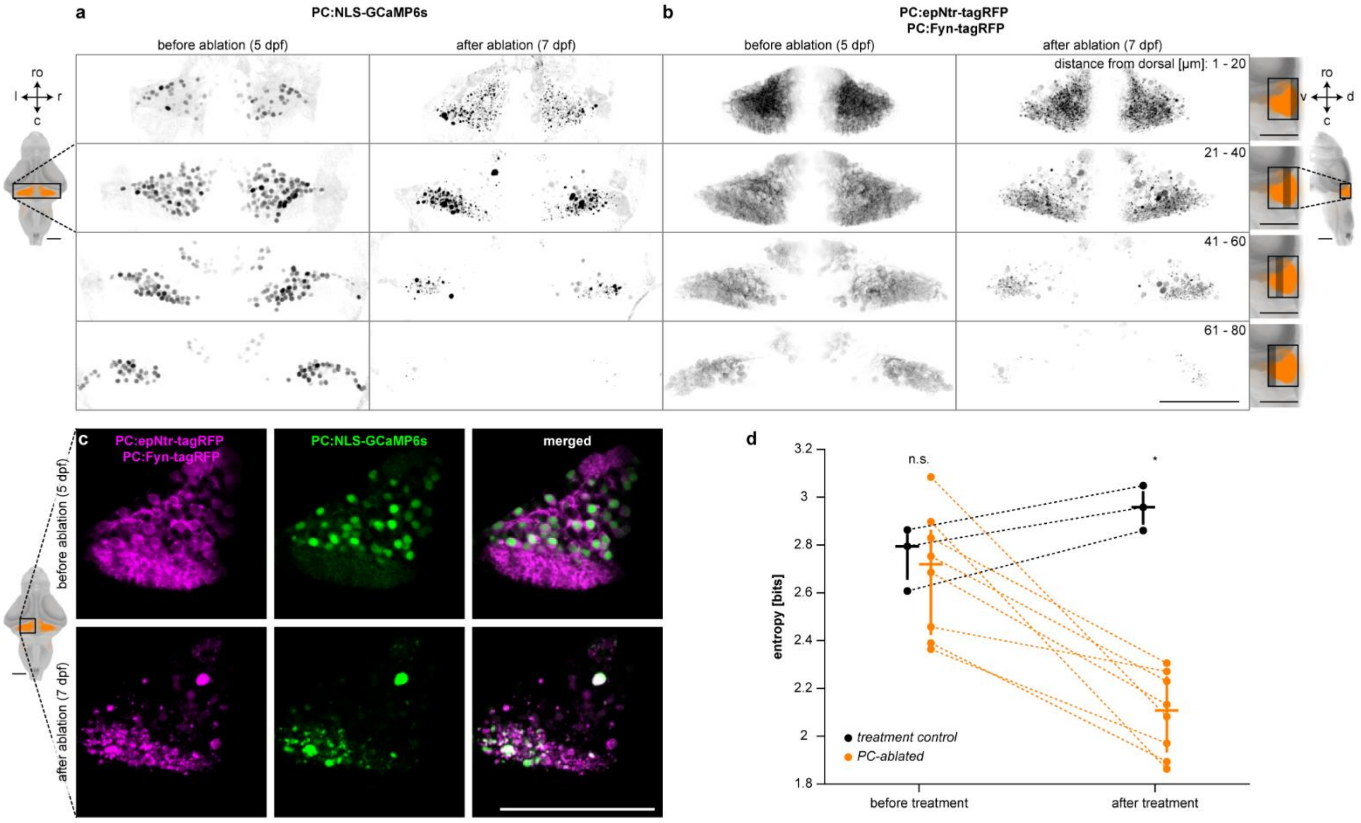

Supplementary Figure 3: Treatment of *Tg(PC:epNtr-tagRFP)* larvae with metronidazole ablates the PCs

Morphology of PC nuclei **(a)** and somata and membranes **(b)** before ablation (5 dpf) and after recovery from the ablation (7 dpf, when the animals' behavior was tested). Each image is a maximum projection of 20 confocal slices, each 1 µm-thick, along the dorsoventral axis in an example larva: ro - rostral direction, l - left, r - right, c - caudal, d - dorsal, v – ventral. Small gray brains illustrate the location of the PCs, shown in orange, within the larval zebrafish reference brain. After ablation, the signal in PCs was much fainter than before, so the contrast of the stack acquired after ablation was manually boosted to visually match the stack obtained before the ablation. In **(a-c)**, scale bars: 100 µm.

(c) Zoomed-in image of one confocal plane (≈ 30 µm from the dorsal surface of the brain).

(d) Local image entropy used to quantify tissue inhomogeneity within the cerebellum before and after ablation. Individual data points, median and interquartile range across larvae are shown (N = 3 *epNtr*$^-$ and 8 *epNtr*$^+$ larvae, n.s. - p = 0.776, * - p = 0.012, Mann-Whitney U test with two-tailed alternative).

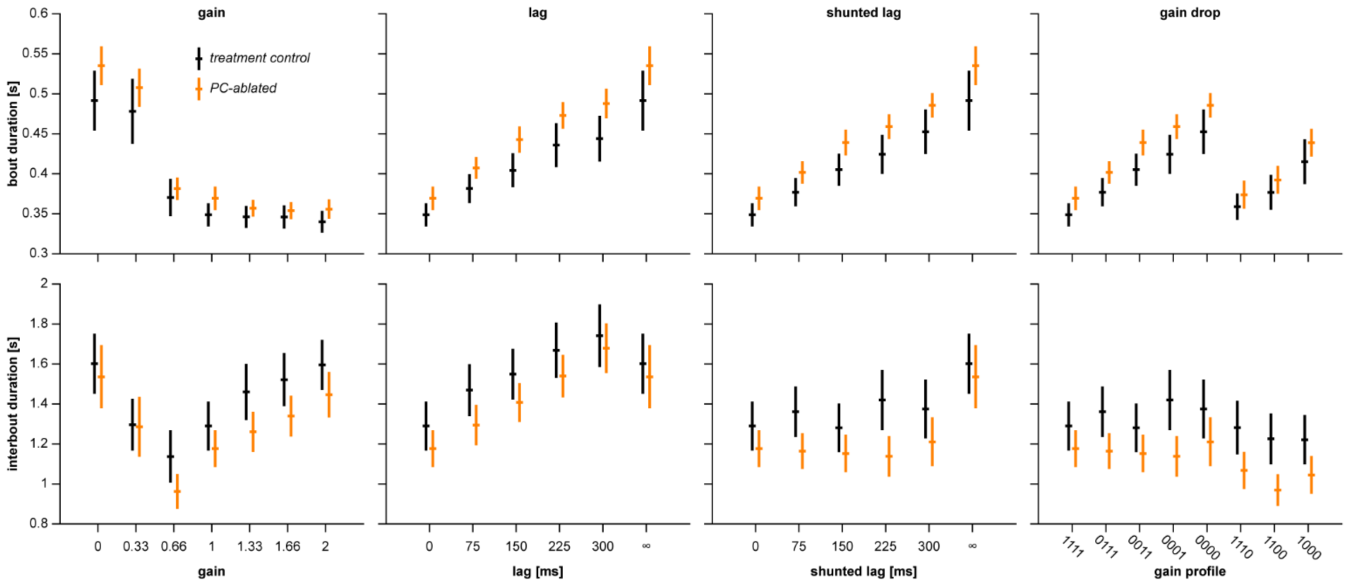

Supplementary Figure 4: Acute reaction is not impaired after PC ablation

Mean bout duration (top) and interbout duration (bottom) in treatment control group (black, N = 28 larvae), and PC ablation group (orange, N = 39 larvae) tested in the acute reaction experiment (Fig. 2) as a function of reafference condition. To obtain data for one larva, all bout and interbout durations were averaged within each reafference condition. Mean ± SEM across larvae is shown. Note that PC-ablated animals demonstrated acute reaction to perturbed visual reafference similarly to the control group.

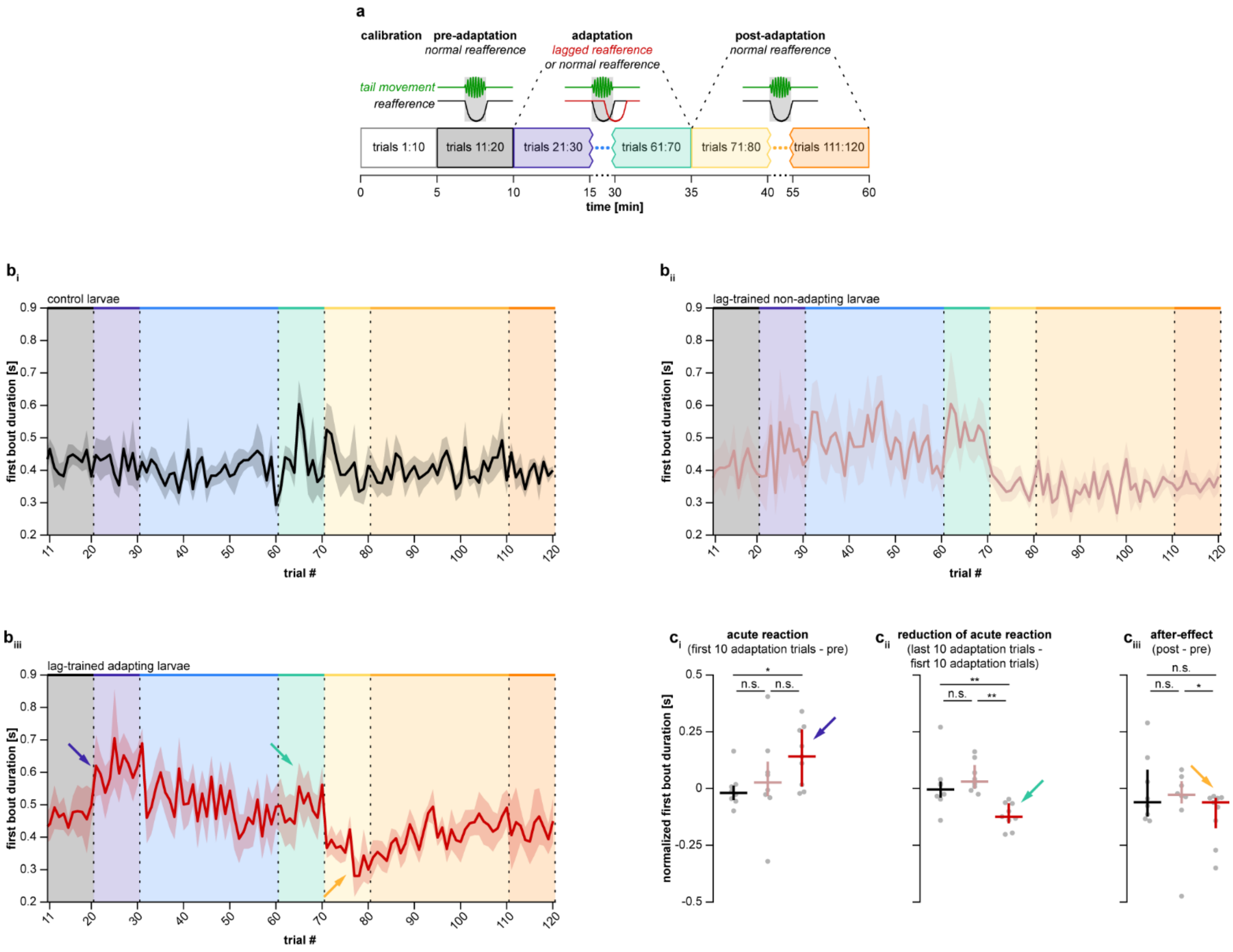

Supplementary Figure 5: Long-term adaptation effects are detectable in the light-sheet functional imaging experiment

(a) Protocol of the long-term adaptation experiment used in the light-sheet experiment (repeated from Fig. 6b for convenience).

(b) First bout duration in each trial in normal-reafference control larvae (i; N = 8), lag-trained non-adapting larvae (ii; N = 8), and lag-trained adapting larvae (iii; N = 9). Solid lines and shaded areas represent mean ± SEM across larvae. Similarly to Fig. 4, blue arrow indicates increase of first bout duration in the beginning of the adaptation phase (acute reaction), cyan arrow indicates decrease of bout duration by the end of the adaptation phase (reduction of acute reaction), and orange arrow indicates decrease of bout duration in the post-adaptation phase (after-effect).

(**c**) Quantification of the acute reaction and long-term adaptation effects. Each gray dot represents first bout duration in one fish, averaged across 10 trials and normalized by subtracting the baseline value obtained during the pre-adaptation phase (i, iii) or during the first 10 trials of the adaptation phase (ii). Black and red lines represent median and interquartile range across larvae; n.s. - $p \geq 0.05$, * - $p < 0.05$ (exact p-values in the following order: control VS non-adapting, control VS adapting, adapting VS non-adapting: **i**: 0.19, 0.01, 0.10, **ii**: 0.92, $0.19 * 10^{-2}$, $4.11 * 10^{-5}$, **iii**: 0.64, 0.24, 0.03; Mann-Whitney U test with one-tailed alternative).

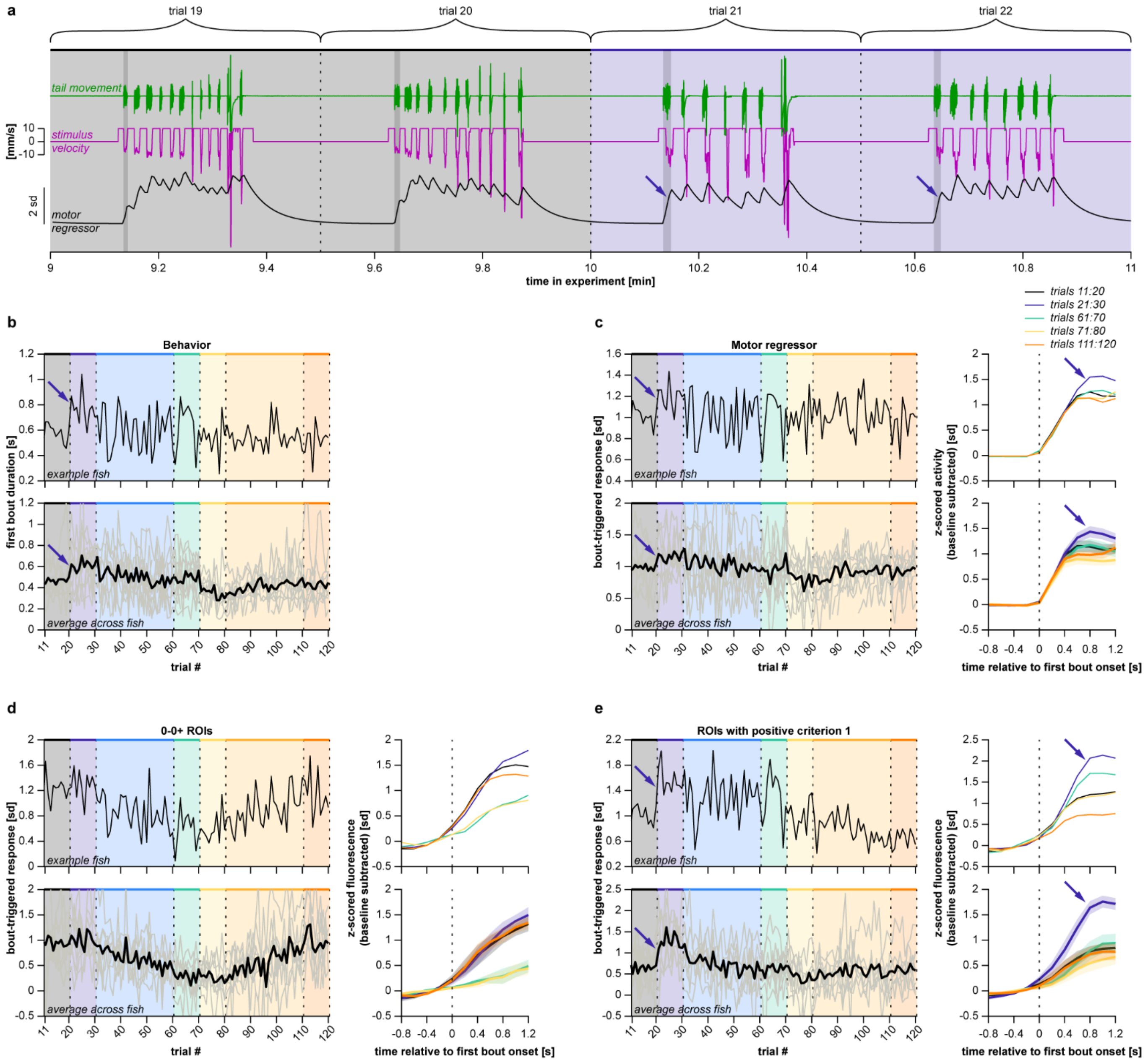

Supplementary Figure 6: Activity of *0-0+* ROIs cannot be explained by behavior

(a) Z-scored activity of a motor regressor that linearly encodes behavior of an example lag-trained larva in four trials. Vertical shaded bars indicate the first swimming bout in each trial. In all panels, color-code for experimental phases is the same as in Fig. 6; blue arrows indicate acute reaction of behavior **(b)** or of bout-triggered responses **(a, c, e)**.

(b) First bout duration in each trial in an example larva shown in **a** (top) and averaged across all lag-trained adapting larvae (N = 9) (bottom). In **b**-**e** (bottom left), thin gray lines represent individual larvae, thick black lines represent mean across larvae.

(c) First-bout-triggered responses of a motor regressor shown in **a** (top left), first-bout-triggered responses of all motor regressors averaged across larvae (bottom left), first-bout-triggered activity of an example motor regressor, averaged across respective blocks of 10 trials (top right), and first-bout-triggered activity of all motor regressors, averaged across respective blocks of 10 trials (bottom right). In **c**-**e** (bottom right), thick colored lines and shaded areas represent mean ± SEM across lag-trained adapting larvae.

(d,e) First-bout-triggered activity of *0-0+* ROIs **(d)** and of ROIs with positive criterion 1 **(e)**. Panels are organized in the same way as in **c**. To compute bout-triggered responses for one larva, we averaged responses of all ROIs from respective cluster detected in that larva.

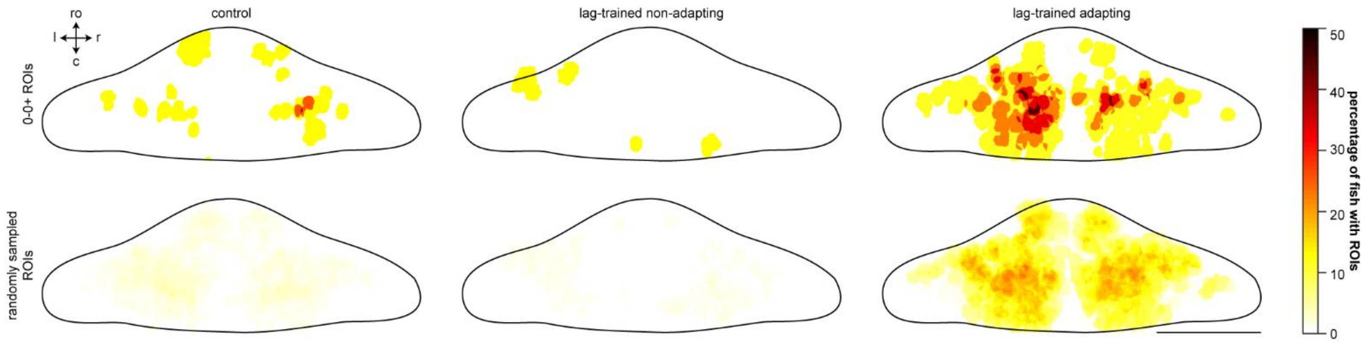

Supplementary Figure 7: ***0-0+*** ROIs represent a spatially distributed subpopulation of PCs

Top, anatomical location of *0-0+* ROIs in normal-reafference control larvae (N = 8), lag-trained non-adapting larvae (N = 8) and lag-trained adapting larvae (N = 9). Color codes for percentage of larvae with ROIs in each voxel of the reference cerebellum. Bottom, anatomical location of randomly sampled ROIs. In each larva, instead of taking *0-0+* ROIs, the same number of random ROIs was sampled. This was repeated 100 times, and presented images are averages across 100 iterations. Note that randomly sampled ROIs in lag-trained adapting larvae appear more often in the medial cerebellum, similarly to *0-0+* ROIs, indicating that this spatial segregation of *0-0+* ROIs is not above the chance level. Scale bar: 100 µm; ro - rostral direction, l - left, r - right, c - caudal.
