# Supplementary material for: A cerebellar internal model calibrates a feedback controller involved in sensorimotor control
Source: Nat Commun. 2021 Nov 18;12:6694. doi: 10.1038/s41467-021-26988-0 (PMC8602262; doi:10.1038/s41467-021-26988-0)
Supplement: Supplementary file 3 — Description of Additional Supplementary Files [file 41467_2021_26988_MOESM3_ESM.pdf]

Description of Additional Supplementary Files

Supplementary Movie 1: Selected reafference conditions used to induce acute reaction

A movie of one trial with seven selected reafference conditions used to induce acute reaction: normal reafference, open loop, gain 0.33, gain 1.66, 225 ms lag, 225 ms shunted lag and gain drop with profile 1100 (see Closed-loop experimental assay in head-restrained zebrafish larvae in Methods for details). These reafference conditions and their order were specifically selected for this video for illustration purposes. Shaded areas indicate swimming bouts. Note that in this movie, the trial duration was 20 s, whereas in all experiments it was 5 s shorter. A longer trial was used in this movie to fit all seven representative reafference conditions into one trial. The video is slowed down by a factor of two.

Supplementary Movie 2: Whole-brain light-sheet imaging in a zebrafish larva performing OMR

A movie of one trial of a light-sheet imaging experiment showing simultaneous behavioral and functional imaging recordings. Left, recorded fluorescence dynamics in one optical plane after alignment (96 µm from the dorsal boundary of the brain) highlighting locations of two example ROIs; ro – rostral direction, l - left, r - right, c – caudal; scale bar, 100 µm. Inset shows an image of the fish tail captured by the behavioral camera and the moving stimulus (grating). Right, stimulus velocity, tail trace and z-scored fluorescence of example ROIs. Shaded areas indicate swimming bouts. Note that the sampling rate of the behavioral data acquisition is much higher than of the functional imaging.

Supplementary Movie 3: Whole-brain light-sheet imaging in a zebrafish larva performing OMR: One full one-hour experiment.

A movie showing the fluorescence responses acquired during an entire one-hour experiment.
