# Supplementary material for: A cerebellar internal model calibrates a feedback controller involved in sensorimotor control
Source: Nat Commun. 2021 Nov 18;12:6694. doi: 10.1038/s41467-021-26988-0 (PMC8602262; doi:10.1038/s41467-021-26988-0)
Supplement: Supplementary file 7 — Reporting Summary [file 41467_2021_26988_MOESM7_ESM.pdf]

Corresponding author(s): Ruben Portugues

Last updated by author(s): Sep 21, 2021

Reporting Summary

Nature Research wishes to improve the reproducibility of the work that we publish. This form provides structure for consistency and transparency in reporting. For further information on Nature Research policies, see our Editorial Policies and the Editorial Policy Checklist.

Statistics

For all statistical analyses, confirm that the following items are present in the figure legend, table legend, main text, or Methods section.

n/a	Confirmed	
☐	☒	The exact sample size (*n*) for each experimental group/condition, given as a discrete number and unit of measurement
☐	☒	A statement on whether measurements were taken from distinct samples or whether the same sample was measured repeatedly
☐	☒	The statistical test(s) used AND whether they are one- or two-sided<br>*Only common tests should be described solely by name; describe more complex techniques in the Methods section.*
☐	☒	A description of all covariates tested
☐	☒	A description of any assumptions or corrections, such as tests of normality and adjustment for multiple comparisons
☐	☒	A full description of the statistical parameters including central tendency (e.g. means) or other basic estimates (e.g. regression coefficient) AND variation (e.g. standard deviation) or associated estimates of uncertainty (e.g. confidence intervals)
☐	☒	For null hypothesis testing, the test statistic (e.g. F, t, r) with confidence intervals, effect sizes, degrees of freedom and P value noted<br>*Give P values as exact values whenever suitable.*
☒	☐	For Bayesian analysis, information on the choice of priors and Markov chain Monte Carlo settings
☒	☐	For hierarchical and complex designs, identification of the appropriate level for tests and full reporting of outcomes
☒	☐	Estimates of effect sizes (e.g. Cohen's *d*, Pearson's *r*), indicating how they were calculated

Our web collection on statistics for biologists contains articles on many of the points above.

Software and code

Policy information about availability of computer code

Data collection	Behavioral data was collected using an open-sourse system for controlling experiments, called Stytra (version 0.8.34). Experimental protocols used in Stytra can be found in the zenodo repository https://doi.org/10.5281/zenodo.5147934, also referred in the Data availibility section. Functional imaging was controlled by custom-written Python software, described in detailed in the Methods section.
Data analysis	Alla data was analyzed using custom-written code, as described in Methods. Behavioral data was analyzed in MATLAB (version R2019a). Imaging data was pre-processed in Python (version 3.7) (scikit-image package (version 0.17.0) was used for data alignment), and further analyzed in MATLAB. All data analysis code can be found in the zenodo repository https://doi.org/10.5281/zenodo.5147934, also referred in the Data availibility section. Furthermore, for computing local entropy of confocal images of the cerebellum before and after ablation, PiPrA software was used (version 0.3.4, https://github.com/anki-xyz/pipra).

For manuscripts utilizing custom algorithms or software that are central to the research but not yet described in published literature, software must be made available to editors and reviewers. We strongly encourage code deposition in a community repository (e.g. GitHub). See the Nature Research guidelines for submitting code & software for further information.

Data

Policy information about availability of data

All manuscripts must include a data availability statement. This statement should provide the following information, where applicable:

- Accession codes, unique identifiers, or web links for publicly available datasets
- A list of figures that have associated raw data
- A description of any restrictions on data availability

The datasets generated and analysed during the current study are available in the zenodo repository, http://doi.org/10.5281/zenodo.5052785. Furthermore, source data are provided with this paper.

Field-specific reporting

Please select the one below that is the best fit for your research. If you are not sure, read the appropriate sections before making your selection.

☒ Life sciences ☐ Behavioural & social sciences ☐ Ecological, evolutionary & environmental sciences

For a reference copy of the document with all sections, see nature.com/documents/nr-reporting-summary-flat.pdf

Life sciences study design

All studies must disclose on these points even when the disclosure is negative.

Sample size

Sample size was determined using previously published studies, including many, but not only, from our lab (Dragomir et al., 2020, Yildizoglu et al., 2020, Portugues et al., 2014). The sample size of the behavioral data set in this study far exceeds any of the sample sizes mentioned above, while the sample size of the imaging experiments is very similar, even though the expeirments outlined here are technically very challenging.

Data exclusions

Behavioral data was excluded based on overall swimming behavior of tested animals. Namely, if there was at least one block of 10 trials with zero swimming bouts, the animal was excluded. The rationale behind this selection was that multiple uncontrolled parameters, such as stress level or damage caused during embedding the agarose, may result in impaired optomotor response. Without this response in the first place, it would be impossible to measure acute and long-term adaptation of it. Functional imaging datasets were excluded in the case of large sample drift during the imaging session, that could not been corrected by our alignment routine. Such drifting samples were identified during manual inspection. They could not be used because our ROI extracting algorythm assumes constant location of each ROI within the field of view throughout the entire session.

Replication

We have successfully reproduced all behavioral findings, including results of Purkinje cell ablation experiment, as well as results of experiments performed on wild-type animals, in three independent datasets, during the pilot experiments aimed to optimise the parameters of the experimental protocols. Both whole-brain and Purkinje cell functional imaging experiments were performed only once after acquisition of the final behavioral dataset, due to their complexity and low throuput.

Randomization

Larvae used for behavioral experiment (both wild-type and PC-ablated) were tested in parallel using four behavioral rigs and three different experimental protocols: acute reaction, long-term adaptation control and long-term adaptation lag. The allocation of animals to each experimental protocol was random. Animals were embedded in the agarose in the morning, and then tested during the day. On each day and on each behavioral rig, the same number of fish were tested in each of the three protocols. This was done to ensure that each clutch and each rig contributes equal number of animals to all three protocols.
Larvae used for PC ablation were screened on 5 days-post-fertilization (i.e. before MTZ treatment) and divided into negative and positive. 10 negative and 10 positive larvae were then re-mixed in one dish to ensure independent sampling and blindness with respect to the experimental group during behavioral testing. Immediately after the experiment, tested larvae were screened again to reasses their genotype.

Blinding

Investigators were blind with respect to experimental group (PC-ablated or treatment control) but not with respect to the experimental protocol (acute reaction or long-term adaptation). Blindness with respect to the experimental group was achieved by mixing positive and negative larvae in one petri dish before MTZ treatment. The genotype was re-assesed after testing under the microscope.
During data analysis, experimentors were not blind with respect to the experimental group. However, this knowledge was not used until the plotting stage (as in truly blind cases) since all datasets were analysed using the same code.

Reporting for specific materials, systems and methods

We require information from authors about some types of materials, experimental systems and methods used in many studies. Here, indicate whether each material, system or method listed is relevant to your study. If you are not sure if a list item applies to your research, read the appropriate section before selecting a response.

Materials & experimental systems

n/a	Involved in the study	
☒	☐	Antibodies
☒	☐	Eukaryotic cell lines
☒	☐	Palaeontology and archaeology
☐	☒	Animals and other organisms
☒	☐	Human research participants
☒	☐	Clinical data
☒	☐	Dual use research of concern

Methods

n/a	Involved in the study	
☒	☐	ChIP-seq
☒	☐	Flow cytometry
☒	☐	MRI-based neuroimaging

Animals and other organisms

Policy information about studies involving animals; ARRIVE guidelines recommended for reporting animal research

Laboratory animals

All experiments were conducted on larval zebrafish (Danio rerio) at 6 - 8 days post-fertilization of yet undetermined sex. Strains used:

Laboratory animals	Tupfel long-fin (TL), Tg(PC:epNtr-tagRFP), Tg(Fyn-tagRFP:PC:NLS-GCaMP6s), Tg(elavl3:GCaMP6s), Tg(PC:-GCaMP6s), Tg(elavl3:GCaMP6f)
Wild animals	The study did not involve wild animals.
Field-collected samples	The study did not involve sample collected from the field.
Ethics oversight	All animal procedures were performed in accordance with approved protocols set by the Max Planck Society and the Regierung von Oberbayern (Protocol number 55-2-1-54-2532-82-2016).

Note that full information on the approval of the study protocol must also be provided in the manuscript.
